# Plant-based biofuels

**DOI:** 10.12688/f1000research.7418.1

**Published:** 2016-02-17

**Authors:** Elizabeth E. Hood

**Affiliations:** 1 College of Agriculture and Technology, Arkansas State University, Arkanas, AR, USA

**Keywords:** Plant-based biofuels, lignocellulosic conversion, biodiesel, biofuels

## Abstract

This review is a short synopsis of some of the latest breakthroughs in the areas of lignocellulosic conversion to fuels and utilization of oils for biodiesel. Although four lignocellulosic ethanol factories have opened in the USA and hundreds of biodiesel installations are active worldwide, technological improvements are being discovered that will rapidly evolve the biofuels industry into a new paradigm. These discoveries involve the feedstocks as well as the technologies to process them.

## Introduction

An important mitigation strategy for the impact of fossil fuels on the environment is to use biofuels from renewable sources for transportation. Biofuels from plants represent the most abundant source of renewable fuels, offering the manufacture of ethanol and butanol (as gasoline additives) and long-chain hydrocarbons (for diesel additives or as jet fuels) from starch, cellulose, hemicellulose, and oils. The source of the energy captured by plants is the sun, which will be a constant source of energy for the next few billion years. The carbon released from the burning of biofuels is continually cycled rather than being released from ancient fixed carbon sources, as is the case for fossil petroleum and natural gas. The problem is that the cost of production of fuels from lignocellulose and plant oils is high and this nascent industry cannot compete with oil prices.

**Figure 1.**
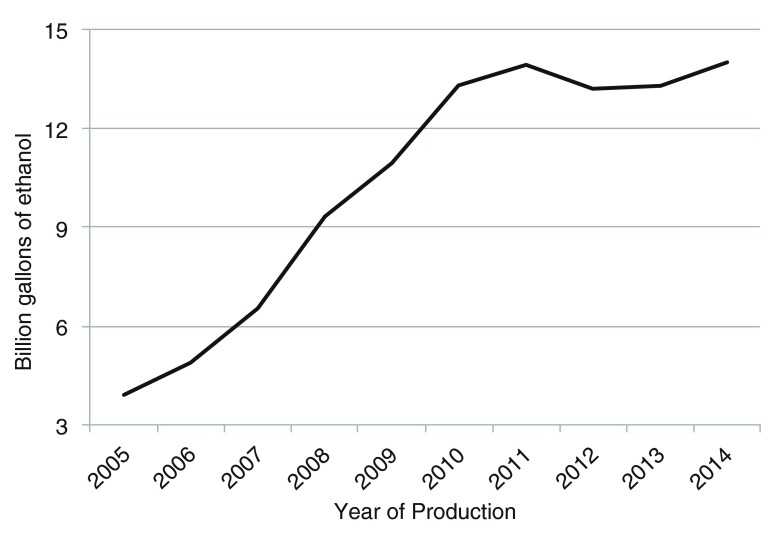
Ethanol production volumes from cornstarch over the last 9 years in the USA. Production has increased from approximately 4 billion gallons (15.1 billion liters) in 2005 to over 14 billion gallons (53 billion liters) in 2014.

*Current progress*: For the past two decades, ethanol has been produced primarily from cornstarch and cane sugar. Fourteen billion gallons of ethanol were produced in the USA from cornstarch in 2014 ( Figure 1). Also shown in Figure 1 is that corn-based ethanol production has plateaued ( http://www.ethanolrfa.org/wp-content/uploads/2015/09/23d732bf7dea55d299_3wm6b6wwl.pdf). Approximately 40% of the current USA corn crop is used to produce ethanol and is not likely to expand anymore, because the remainder of the crop is being used for animal feed and human food. Ethanol is produced from cane sugar in Brazil at a level of 7.3 billion gallons in 2014 ( http://sugarcane.org/sugarcane-products/ethanol). Together, Brazil and the USA produce more than 90% of the world’s supply of ethanol.

**Figure 2.**
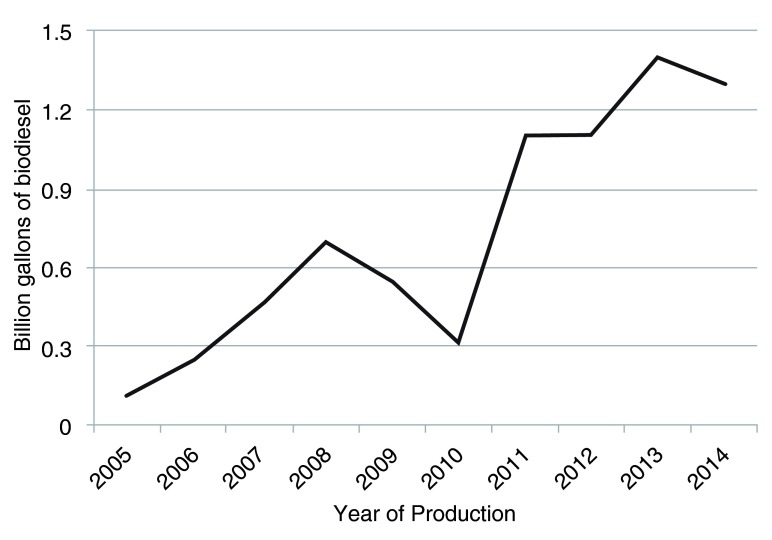
Biodiesel production volumes from all source oils over the last 9 years in the USA. Production has increased from 112 million gallons in 2005 to over 1.3 billion gallons in 2014. A large dip in production was seen from 2008 to 2010 during the economic recession.

Biodiesel is a renewable fuel that has received considerable attention recently because it is also non-polluting. It is carbon neutral because the carbon present in vehicle exhaust was recently fixed from atmospheric carbon ^1^. Biodiesel can be manufactured from numerous oils and fats including virgin vegetable oils, such as canola, soybean, and camelina, from waste cooking oils, or from animal fats, such as tallow or lard. The global biodiesel industry has grown considerably over the last several years ^2^, although since 2008 a dip occurred based on world economic recession. Europe has produced the greatest volume of biodiesel over the years, followed by the USA. Worldwide production in 2012 comprised 6 billion gallons (22.5 billion liters) ( http://www.uabio.org/img/files/docs/140526-wba-gbs-2014.pdf). Production in the USA in 2012 was approximately 0.89 billion gallons but increased to over 1.25 billion gallons in 2014 (Energy Information Administration as shown in Figure 2).

*The Renewable Fuel Standard II (RFS II)*: RFS II is the motivation for increasing the production of renewable fuels from green plants ( http://www.ethanolrfa.org/policy/regulations/renewable-fuel-standard/). This standard was set in 2005 and revised in 2007 to mandate quantities of renewable fuels to be incorporated into the transportation industry in the USA. The goal for 2022 is set at 36 billion gallons of renewable fuels, with 16 billion gallons required to be from lignocellulosic feedstocks, and 1 billion gallons per year of biodiesel. Additionally, 58% of the fuels produced by 2022 should be “advanced biofuels”, e.g. non-starch ethanol or other types of fuels such as long-chain hydrocarbons or butanol that achieve a 50% reduction in greenhouse gas emissions. The feedstocks for these fuels are lignocellulose and oils. However, intense research is necessary to make this cost-competitive. Breakthroughs are being made in feedstock structure, ease of processing, efficiency of conversion, co-product manufacture, and sustainability. As these discoveries come together, they can be incorporated into new industrial applications.

## Lignocellulosic biofuels

**Figure 3.**
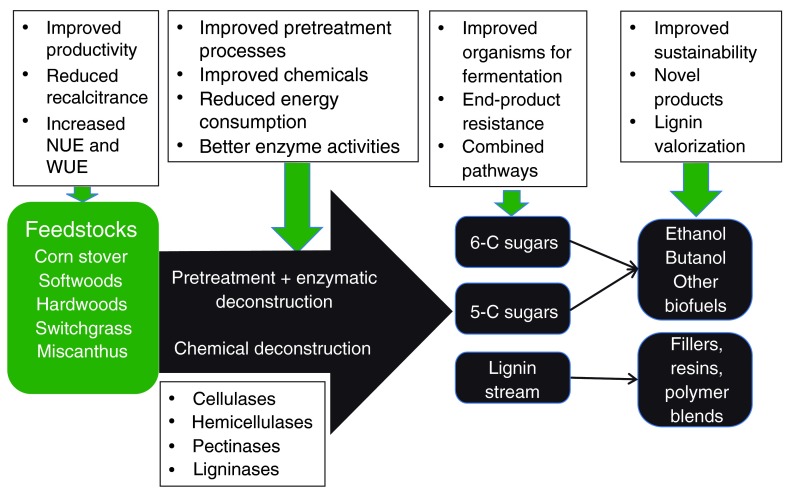
Production of biofuels from cellulosic feedstocks. A variety of feedstocks can be harvested, ground, and fed into unit operations that allow the tight cell wall structures to be loosened (pretreatment) for enzymatic deconstruction, or for chemical deconstruction to be accomplished. Resulting chemicals comprise five- and six-carbon sugars and lignin in a range of polymerization degrees (monomers to long polymers). Biofuels and biobased products are manufactured from these raw materials through a variety of microbial or chemical conversion processes. Research in each of these areas is highlighted in the upper boxes and discussed in the review.

*Research Roadmap*: In 2007, the US Department of Energy conducted a workshop to assess the major roadblocks to the production of lignocellulosic biofuels ( https://www1.eere.energy.gov/bioenergy/pdfs/obp_roadmapv2_web.pdf). The purpose of the workshop was to guide research and development activities that would enable the biofuels industry in an accelerated time frame. Barriers were identified for feedstocks, deconstruction and conversion, infrastructure, and sustainability of the industry. In the case of feedstocks, yield of biomass, broader feedstock variety base, reduction of recalcitrance, and improved nutrient and water use efficiency were targets ( Figure 3). For deconstruction and conversion to biofuels, reducing the cost of enzymes and improving conversion efficiencies were targets to reduce cost, as well as broadening the number and condition of crops that could be used as feedstock. An overlying goal is to improve the sustainability of the industry, from crop growth and harvest through to product manufacture and feedstock utilization. Although infrastructure and outreach were part of the roadmap, they will not be addressed in this review. This Roadmap was used to establish a call for proposals to fund three Bioenergy Research Centers, resulting in the Great Lakes Bioenergy Research Center, the BioEnergy Science Center, and the Joint BioEnergy Institute. These three centers along with numerous other smaller entities are making significant progress toward addressing these barriers through targeted research programs.

*Feedstock recalcitrance and productivity*: The first four lignocellulosic biofuel manufacturing plants have been opened in the USA. Their biomass feedstocks comprise residual materials from food crops and forestry operations. Two installations in Iowa owned by Poet, LLC (Sioux Falls, SD) and DuPont (Wilmington, DE) use corn stover and residual materials as their feedstock. The Abengoa Bioenergy Biomass of Kansas (ABBK) installation in Hugoton, KS, used primarily wheat straw (the plant is now closed and is for sale, at time of writing), although other feedstocks were used as available. INEOS Bio in Vero Beach, FL, utilizes vegetative and wood waste. The next generation of biofuel installations likely will use dedicated biomass crops such as poplar, switchgrass, sorghum, and miscanthus, because they are in intense research programs to improve their yield and digestibility.

Lignin is the primary molecule that contributes to recalcitrance (lack of digestibility) because of its complex structure and prevalent ether bonds that are difficult to break. It is also cross-linked to many potentially digestible cell wall polymers ^3^. A recent breakthrough in lowering recalcitrance is to engineer plants to have different linkages in lignin ^4– 7^. The enzyme monolignol ferulate transferase introduces chemically labile linkages into the lignin backbone, facilitating the release of five- and six-carbon sugars from cell wall polysaccharides after mild pretreatment ^6^. This Zip Lignin™ ( https://www.glbrc.org/research/technologies/grass-modified-easier-bioprocessing) has multiple ester linkages that are far easier to break than the more common ether linkages ^6^. Down-regulation of caffeic acid O-methyltransferase (COMT) in switchgrass also lowers lignin ^8^. Although only a modest amount lower, these plants have a reduced syringyl:guaiacyl lignin monomer ratio and increased ethanol yield by up to 38% using conventional biomass fermentation processes ^8^.

Several other cell wall mutations or manipulations have increased productivity of biomass and/or lowered biomass recalcitrance. Enhancing syringyl lignin in pine tracheary elements could enhance bioprocessing ^9^, enhanced content of sinapaldehyde in lignin in Arabidopsis leads to enhanced digestibility of cell walls ^10^, and adding bacterial genes into Arabidopsis can form oxidized lignin that is more easily digested ^11^. Lowering total lignin content rather than altering monomer ratios through manipulations of the p-coumaroyl quinate/shikimate 3′-hydroxylase and cinnamate 4-hydroxylase genes in *Eucalyptus* significantly lowered recalcitrance ^12^. Baxter and colleagues found that over-expression of the switchgrass transcription factor PvMYB4, which acts as a transcriptional repressor of many lignin biosynthetic genes, reduced lignin in transgenic switchgrass plants by as much as 50% ^13^. While some of the transgenic plants were less field-hardy, one robust transgenic line had 63% greater biomass and yielded 32% more biofuel.

In efforts to understand pectin synthesis and accumulation, galacturonosyl transferase (GAUT) gene family members were over- and under-expressed in switchgrass. Under-expression through RNA interference (RNAi) of GAUT12.1 lowered pectin content and surprisingly increased glucose release by as much as 8% over controls without compromising growth, actually increasing plant height and stem diameter ^14, 15^. These knockdown mutants had less xylan as well as less pectin, although total lignin content was the same as in controls.

In addition to manipulating cell wall recalcitrance, plants have been manipulated to exhibit traits that increase biomass production. For example, *Brachypodium distachyon* plants in which phytochrome C is down-regulated have greatly delayed flowering, contributing to enhanced biomass accumulation ^16^. Quantitative trait loci (QTLs) were identified in switchgrass that define biomass yield and plant height ^17^. QTLs are groups of genes that co-segregate and act together to generate the phenotype of interest.

*Deconstruction and fuel production*: A number of different pretreatment regimes have been explored in detail to be used with various enzyme systems. Ammonia fiber expansion (90–120°C; 250–400 psi) is highly effective on grasses such as corn stover and switchgrass but does not produce a lignin fraction that can be used for co-product manufacture ^18^. Lignin is desirable because its monomers can be used as a raw material for many more complex chemicals. Because the ammonia can be quantitatively recovered, it is relatively cost effective. Alkaline hydrogen peroxide is used in pulp-bleaching and is an effective delignification agent ^19^. However, the high concentration of hydrogen peroxide made this method cost-prohibitive. The addition of a small amount of copper ions significantly improves the lignin extraction, thus making it highly effective for woody biomass pretreatment ^20^. These treatments usually are followed by treatment with enzyme mixtures to deconstruct the cellulose and hemicellulose from the cell walls, producing sugars for fermentation, though in order for the enzymes to function, the biomass alkalinity must be neutralized, adding further cost to the processing and generating additional waste.

A promising new solvent for treating biomass is γ-valerolactone, or GVL ^21^. It is derived from the biomass itself and appears to pretreat any type of biomass ^22^ yielding sugar, lignin, and mineral salt streams that can be separated from the reaction mix ^23^. Ionic liquids (ILs) offer a pretreatment strategy that has many advantages, including significant enhancement in the rate of enzyme hydrolysis of the cellulose component of switchgrass and a 96% recovery of glucan in 24 hours ^24^. However, IL is quite expensive at its current state of development.

Pretreated biomass is generally deconstructed with enzymes, whether these are mixtures isolated from fungal cultures, multifunctional enzymes isolated from microorganisms, or mixtures isolated from live bioprocessing organisms growing on the biomass. To use enzymes cost-effectively for biomass conversion prior to fermentation, it is estimated that the cost of the enzymes should be approximately $0.10 per gallon of ethanol ^25, 26^. For the past 15 years, intense research on enzyme production platforms has yielded fungal enzyme mixtures that do not meet these cost requirements and in fact also require a huge infrastructure for production. A relatively new technology utilizes genetically engineered plant seeds (primarily maize) to accumulate industrial enzymes ^25^. The plant seed production system is more cost-competitive but has not been tested at scale for efficacy ^25^. Current research efforts are in multifunctional enzymes (USP application # 2014/0079683) and combined bioprocessing organisms, the latter of which can decompose plant polymers as well as ferment them into biofuels ^27^.

Once the biomass has been deconstructed into sugars, those sugars should be fermented into fuels such as ethanol, butanol, or longer-chain hydrocarbons. These products are currently manufactured through bacterial ( *Clostridium* sp.) or yeast fermentation. However, new microorganisms, many of which are thermophilic bacteria, are under investigation as potential biofuel-producing microbes ^27^. Significant changes in their metabolic networks are required to allow them to produce a single product, such as ethanol, at high titer and without inhibition by the product. Additional inhibition is seen as a result of pretreatment of various lignocellulosic feedstocks ^28^. These thermophiles, such as *Caldicellulosiruptor bescii*, can also be used to probe plant cell wall structure through mutated enzyme activities ^29^.

*Sustainability*: Production of biomass for non-food uses, such as for biofuels and biobased products, has faced a fierce debate with advocates for food production on arable agricultural land, as well as land use changes. Thus, research on increasing the productivity of biomass crops, use of marginal lands for production, fewer inputs such as water and nutrients, and recovering maximal sugars and co-products are key outcomes for achieving the goal of production of billions of gallons of biofuels from lignocellulosic feedstock. Life cycle assessments of various biomass crops, produced in different soils under different environmental conditions, are being completed to understand the best cropping systems with the best environmental outcome ^30^. In addition, the square footage of the biorefinery that produces the fuel can have a major impact on sustainability, impacting the transportation radius for bringing biomass from the surrounding area ^31^. Growth and nutrient-use advantages are being seen through inoculation with endophytic fungi ^32^, and manipulation of transcription factor genes that are important for nutrient cycling during senescence may improve nutrient-use efficiency in perennial plants such as switchgrass ^33^.

## Plant oils to biofuels

**Figure 4.**
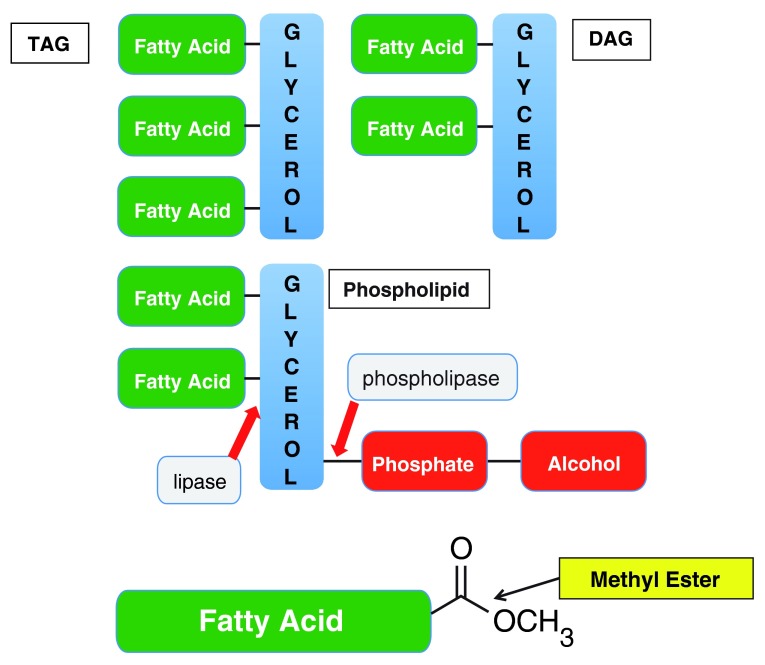
Structures of precursors and product for biodiesel. Top: TAG (triacylglycerol); DAG (diacylglycerol); center: phospholipid; bottom: fatty acid methyl esters (FAMEs) or biodiesel produced through trans-esterification. Phospholipase yields DAG and R-phosphate. Lipase yields FAMEs.

*Biodiesel-esters from triacylglycerol (TAG) and diacylglycerol (DAG, Figure 4)*: Biodiesel is predominantly commercially manufactured through treatment of feedstock oil or fat with alcohols and chemical catalysts such as sodium or potassium hydroxide. Although this method can be commercially profitable, this is true only when certain conditions are met ^1, 34, 35^. First, virgin plant oils are generally too expensive for the process and the more cost-effective feedstock is usually recycled cooking oil or other used oils ^2^. Second, the feedstock oil must be low in free fatty acids (FFAs, less than 2%), or those FFAs must be removed prior to chemical reactions. If not removed, they saponify (i.e. make soaps), forming emulsions with the catalyst, and make the biodiesel more difficult to separate from the contaminants ^36^. Third, phospholipids must also be removed because they result in foaming or emulsions, which are difficult to remove from the finished biodiesel. Additional considerations are that the process is energy intensive and the alkaline waste water requires treatment for reuse ^2^. The primary products of fats and oils are fatty acid methyl esters (FAMEs, biodiesel, Figure 4) ^35^, which must be separated from the contaminating glycerol, water, and acid or base catalyst.

Using enzymes rather than non-enzymatic catalysts can address several of these issues. Biological processes in any type of industrial reaction can promote more specific products from the feedstock and can avoid co-product formation ^2^. Additionally, the products are regiospecific, i.e. the enzyme selectively generates one constitutional isomer rather than the other. Moreover, they represent an environmentally friendly process for making biodiesel that yields methyl esters from FFAs and from the DAG liberated by phospholipases from phospholipids, increasing the overall biodiesel yield from the feedstock oil.

*Lipases and phospholipases*: Lipases (EC 3.1.1.3) are carboxylesterases, the most important group of biocatalysts for biotechnological applications in organic chemistry ^37^. Numerous lipases have been identified that are active on various substrates to a greater or lesser degree. Commercial lipases are currently produced through microbial fermentation and used industrially for the synthesis of biopolymers, biodiesel, agrochemicals, flavor compounds, and enantiopure pharmaceuticals. They catalyze highly stereospecific reactions and have very few side reactions. For biodiesel application, large volumes of low-cost enzymes that can be immobilized onto appropriate substrates will enable enzymatic biodiesel production on a large scale. Phospholipase, in particular phospholipase C (PLC, EC 3.1.4.3), catalyzes the hydrolysis of phosphate-R groups from phospholipids ( Figure 4), resulting in DAG. DAG is an excellent substrate for the lipases in trans-esterification reactions, improving the yield of biodiesel from oil feedstocks. The enzyme has been produced commercially in *Pichia pastoris* ( http://www.fao.org/fileadmin/templates/agns/pdf/jecfa/cta/69/Phospholipase_C.pdf).

*Oil degumming using phospholipase*: Degumming is an essential process for purifying waste cooking and vegetable oils to produce a quality feedstock for biodiesel ^38^. The process removes the contaminating phosphorus-containing lipids ( Figure 4), which act as emulsifiers and trap neutral oil, resulting in loss of biodiesel feedstock. For chemically catalyzed biodiesel, the phospholipid content of the oil must be less than 10 parts per million (ppm) and is often removed from the feedstock by mixing a small volume of water with the oil. The majority of these aliphatic phospholipids form a gummy mass that can be removed by centrifugation or filtration. Phospholipase A _1_, A _2_, or C can also be used to remove phospholipids. The enzyme releases 1,2-DAG that can be processed through either enzymatic or chemical processes to FAMEs.

*Lipase can produce FAME through esterification and trans-esterification*: The primary reaction to produce biodiesel is to convert di- and tri-glycerides ( Figure 4) into methyl esters. Biodiesel comprises these FAMEs. Chemical conversion uses a large excess of methanol to push the reaction toward the product and its co-product glycerol ^36^. Using lipase allows for stoichiometric and slow addition of methanol to the reaction mixture because the enzyme itself pushes the reaction toward the regiospecific product.

## Conclusions

Plants as sources of biofuels have many advantages, particularly a neutral carbon balance. Although much rhetoric has surfaced to discourage the growth of biofuel crops because they utilize farmland that should be dedicated to food crops, in reality the productivity and yield of fuels from dedicated energy crops appears to be on a steep, upward trajectory. Moreover, the technology to produce ethanol and biodiesel from plant biomass has progressed at a phenomenal rate, generating confidence that the industry will be profitable and sustainable. Because biomass varies widely in chemical composition and structure, and processes applicable to each biomass type vary as well, smaller biorefineries may be the standard of this industry rather than requiring large biorefineries to reach economic feasibility ^31, 39^. This conclusion is based on the cited studies that look at specific processes applied to specific biomass types, such as woody biomass and GVL, which would enable a biorefinery to produce fuels efficiently from a specific biomass type. Although not discussed here, a contributing factor to the profitability of the industry will be the manufacture of co-products from the biomass: carbon fibers, fillers, resins, or polymer blends from lignin ^22, 40, 41^ or pre-extracted plastics ^42^ or enzymes ^43^ prior to deconstructing the biomass.

## Abbreviations

DAG, Diacylglycerol; FAME, Fatty Acid Methyl Ester; FFA, Free Fatty Acids; GAUT, Galacturonosyl transferase; GVL, γ-valerolactone; IL, Ionic liquid.
